# Differences in insulin sensitivity, lipid metabolism and inflammation between young adult Pakistani and Norwegian patients with type 2 diabetes: a cross sectional study

**DOI:** 10.1186/1472-6823-13-49

**Published:** 2013-10-22

**Authors:** Cecilie Wium, Erlend T Aasheim, Thor Ueland, Annika E Michelsen, Per M Thorsby, Ingegerd F Larsen, Peter A Torjesen, Pål Aukrust, Kåre I Birkeland

**Affiliations:** 1 Hormone Laboratory, Oslo University Hospital, Oslo, Norway; 2 Department of Endocrinology, Morbid Obesity and Preventive Medicine, Oslo University Hospital, Oslo, Norway; 3 Faculty of Medicine, University of Oslo, Oslo, Norway; 4 Research Institute of Internal Medicine, Oslo University Hospital, Oslo, Norway; 5 Department of Medicine, Lovisenberg Deaconess Hospital, Oslo, Norway; 6 Section of Clinical Immunology and Infectious Diseases, Oslo University Hospital, Oslo, Norway

**Keywords:** Ethnicity, South Asian, Insulin sensitivity, Anthropometry, NEFA, Adipokines, Inflammation

## Abstract

**Background:**

Immigrants from South Asia to Western countries have a high prevalence of type 2 diabetes mellitus (T2DM). We explored pathogenic factors that might contribute to the high risk of T2DM in Pakistani immigrants to Norway.

**Methods:**

A cross-sectional study was performed in 18 Pakistani and 21 Norwegian men and women with T2DM (age 29 – 45 years), recruited from two hospital out-patient clinics. Anthropometrics and a two-step euglycemic, hyperinsulinemic clamp with measurements of non-esterified fatty acids (NEFA) during clamp, was performed in all patients. Insulin sensitivity, given as the Glucose Infusion Rate (GIR) and Insulin Sensitivity Index (ISI), was calculated from the two euglycemic clamp steps. Fasting adipokines and inflammatory mediators were measured. Continuous variables between groups were compared using Student’s *t* test or Mann–Whitney *U* test as appropriate. Spearman’s correlation coefficient and multiple linear regression analyses were used.

**Results:**

Despite having a lower BMI, Pakistani patients were more insulin resistant than Norwegian patients, during both low and high insulin infusion rates, after adjustment for sex and % body fat: median (interquartile range) GIR_(low insulin)_: 339.8(468.0) vs 468.4(587.3) μmol/m^2^/min (p = 0.060), ISI_(low insulin)_: 57.1(74.1) vs 79.7(137.9) μmol/m^2^/min (p = 0.012), GIR_(high insulin)_: 1661.1(672.3) vs 2055.6(907.0) μmol/m^2^/min (p = 0.042), ISI_(high insulin)_: 14.2(7.3) vs 20.7(17.2) μmol/m^2^/min (p = 0.014). Pakistani patients had lower percentage NEFA suppression 30 minutes into clamp hyperinsulinemia than Norwegians: 41.9(90.6)% vs 71.2(42.1)%, (p = 0.042). The relationship of ISI to BMI, leptin and interleukin-1 receptor antagonist also differed between Norwegians and Pakistanis**.**

**Conclusions:**

Compared with Norwegian patients, Pakistani patients with T2DM had lower insulin sensitivity, affecting both glucose and lipid metabolism. The relation of insulin sensitivity to BMI and some adipokines also differed between the groups.

## Background

Immigrants from South Asia to Western countries have a high prevalence of Type 2 diabetes mellitus (T2DM) [1-4]. The prevalence is also high in urban areas in their countries of origin [5-7]. Studies comparing non-diabetic subjects from India and the US indicate that Indians have lower insulin sensitivity [8,9]. Norway, like many other Western countries, has a growing population of immigrants, with one of the largest groups being of Pakistani origin. This group has an alarming prevalence of T2DM, manifesting at a younger age than in ethnic Norwegians [10], and reaching more than 25% in 30–60 year old South Asian women living in an Eastern suburb of Oslo [11]. Better knowledge through studies of the pathophysiology of T2DM in this population is necessary to develop more efficient prevention and treatment strategies. Such studies could also give new insight into the pathogenesis of T2DM in general.

There is a well known association between obesity and T2DM, but South Asians develop T2DM at lower levels of body mass index (BMI) than Westerners, and are more insulin resistant for any given BMI [12,13]. Several studies have indicated that, in individuals from South Asia, BMI may be inferior to other measures of adiposity as a predictor of metabolic risk [13-15].

Adipokines and inflammatory mediators derived from adipose tissue may be important in the pathogenesis of insulin resistance and T2DM [16]. Possible differences in the profile of these substances between South Asians and Westerners with T2DM, could potentially help explain differences in the development of T2DM in these groups.

In the present study, we investigated a group of young adult immigrants from Pakistan with T2DM, and compared them to a group of Norwegian patients of similar age. We chose to study young adult subjects, where disease duration was relatively short, to avoid comorbidities that might confound the interpretation of our data. Our aim was to explore differences between the groups with regards to pathogenic factors such as insulin resistance, obesity and inflammation, which might contribute to the high prevalence of T2DM in Pakistani immigrants to Norway.

## Methods

### Design

This was a cross-sectional study comparing young T2DM patients from two different ethnic groups.

### Patients

Norwegian and Pakistani patients with T2DM, aged 45 years or younger, attending the out-patient clinics at Lovisenberg Deaconess Hospital between 1999 and 2005, and Aker University Hospital between 2003 and 2009,were eligible for inclusion. After searches in the two hospitals’ patient registries, 195 patients were randomly selected to receive an invitation to participate in the study. Exclusion criteria were: ethnicities other than Norwegian or Pakistani, positive GAD or IA2 auto-antibodies, age > 45 years, person unwilling or unable to give informed consent. Patients were contacted by letter with information about the study in Norwegian and Urdu, and patients whose telephone number could be obtained were also informed by phone. Nineteen Pakistani and 21 Norwegian sex-matched patients (age 29–45 years, 49% men) with confirmed T2DM, agreed to participate, and were included in the study. Participant characteristics were similar to a subgroup of approximately 80 of the 155 patients not included, where data on HbA_1c_ and anthropometrics were available. All Pakistani participants were first generation immigrants. One Pakistani woman was excluded on the first day of testing, because of difficulties in obtaining intravenous access. The remaining 39 patients were examined.

### Anthropometrical measurements

Height and weight were measured with participants wearing light clothing and without any shoes on. Waist and hip circumferences were assessed with a tape measure at mid point between the lowest rib margin and the iliac crest, and at the level of the major trochanter, respectively. Bioelectrical impedance analysis (BIA) was performed on a Tanita Body Composition Analyzer BC-418 (Tokyo, Japan). All subjects were fasting and voided urine before measurement.

### Euglycemic clamp

To enhance comparability of examinations, all patients stopped taking oral antidiabetic drugs for two days, and insulin for at least 12 hours prior to examination. Patients were asked to refrain from strenuous physical exercise and alcohol intake during these two days, and to fast from midnight during the night before the examination. We performed a euglycemic, hyperinsulinemic clamp, using a modification of the method originally described by De Fronzo et al. [17]. The clamp was performed with two steps, first administering a primed, continuous insulin dose of 40 mU/m^2^/min for a minimum of 100 minutes, until at least 30 minutes of stable euglycemia was obtained. This was directly followed by a 400 mU/m^2^/min insulin infusion, also for a minimum of 100 minutes, with at least 30 minutes of stable euglycemia at the end. The body surface area was calculated using Mostellers equation [18].

Human insulin (Actrapid®, Novo Nordisk, Bagsvaerd, Denmark) 300 mU/mL and Glucose 200 mg/mL were infused through a teflon catheter in a vein at the left elbow of the patient. Insulin was diluted in NaCl 0.9%, after having first added 2 ml of the patients own blood, to avoid insulin sticking to the walls of the bag. All blood samples were drawn from a teflon catheter in a vein at the right elbow, kept open by a slow infusion of NaCl 0.9%. The right arm was kept at 37°C by a heating sleeve connected to a thermal control unit (Swetron AB, Veddestad, Sweden), to arterialize blood samples. Plasma glucose was measured every five minutes using a OneTouch Ultra glucose measuring device (LifeScan, Milpitas, CA), with control measurements every 30 minutes by the glucose oxidase method, on a Glucose Analyzer II (Beckman Instruments, Fullerton, CA). At the end of each step of the clamp, three measurements of serum insulin were taken at ten-minute intervals. The glucose infusion rate in μmol/m^2^/min was established, and denoted GIR_40_ and GIR_400_ respectively. Because of varying metabolic clearance rates of insulin, the insulin levels measured at the end of each step of the clamp differed between patients. The insulin sensitivity index (ISI) was therefore also calculated, and expressed as the ratio of the GIR to the prevailing mean serum insulin levels ((GIR/I) × 100), denoted ISI_40_ and ISI_400_. Every 30 minutes during the 2 step euglycemic clamp, EDTA plasma for non-esterified fatty acid (NEFA) measurements was extracted and immediately frozen at -70°C.

Two Norwegian and two Pakistani patients did not attain euglycemia during the first step of the clamp, and were excluded from this part of the clamp analyses. Two Norwegian and four Pakistani patients were not examined with the high-step clamp, due to contraindication of hyperosmolar glucose at high infusion rates (for the most insulin sensitive patients) and long duration of the low-step part of the clamp to reach euglycemia (for the most insulin resistant patients). After excluding these patients from clamp analyses, there were 46% men in the first step and 48% men in the second step of the clamp.

### Blood samples

Fasting plasma glucose was measured by the glucose oxidase method on a Glucose Analyzer II (Beckman Instruments). Serum insulin was analyzed using the radioimmunoassay (RIA) kit, formerly from Linco Research Inc. (St. Charles, MO), presently available from Millipore Corp. (Billerica, MA). Plasma HbA_1c_ was measured by high performance liquid chromatography on a Variant analyzer (Bio-Rad, Richmond, CA). Fasting serum total cholesterol, serum HDL cholesterol, and serum triglycerides were measured using a routine enzymatic method (Roche Diagnostics, Mannheim, Germany). Serum LDL cholesterol was calculated using the Friedewald equation [19]. NEFA were analyzed using a NEFA C enzymatic color test kit, (Wako Chaemicals GmbH, Neuss, Germany), modified to run on a Technicon RA1000 (Technicon Instruments Corp., Tarrytown, NY). Plasma levels of adiponectin and leptin were analyzed using RIA kits from Millipore Corp. (Billerica, MA),(also formerly from Linco Research Inc. (St. Charles, MO)). Plasma measurements of soluble tumor necrosis factor-receptor type 1 (sTNF-R1) and high sensitive C-reactive protein (hsCRP) were performed using DuoSet ELISA kits from R&D Systems (Minneapolis, MN). Plasma measurements of interleukin-1 receptor antagonist (IL-1RA) were performed using CytoSet from Invitrogen Corporation (Carlsbad, CA), with streptavidin-horseradish peroxydase from R&D Systems. Plasma measurements of interleukin-6 (IL-6) were performed using a High Sensitivity ELISA kit from Abcam plc. (Cambridge, UK).

### Statistical analysis

Data are presented as mean ± SD, or median (interquartile range) as appropriate. We analysed non-normally distributed data log-transformed, or with the use of non-parametric methods. Student’s *t* test or Mann Whitney U tests were used for comparison of continuous variables between groups. For correlations, Spearman’s correlation coefficient (r_s_) was used. Multiple linear regression analyses were performed with log-transformation of parameters when needed, to obtain normally distributed residuals. A two-sided p-value <0.05 was considered significant, but owing to the large number of comparisons, particular attention should be directed towards analyses where p-values are <0.01. Statistical analyses were performed with SPSS 18.0 for Windows (SPSS Inc., Chicago, IL).

### Ethics

The study was carried out in accordance with the Helsinki Declaration, and approved by the Eastern Norway Regional Committee for Medical and Health Research Ethics. Informed, written consent was obtained from each participant before enrolment.

## Results

### Ethnic differences in diabetes duration and anthropometrics

The two ethnic groups did not differ significantly in age, sex and fasting plasma glucose. In contrast, BMI and weight, including both total body fat mass and lean body mass were significantly higher in the Norwegian, compared with the Pakistani group (Table 1). Despite lower BMI, Pakistani patients had higher median HbA_1c_, longer median duration of diabetes, and a tendency towards earlier onset of diabetes (reported mean age at onset 30 years vs 34 years, *p = 0.06*). Further clinical characteristics of the participating patients are presented in Additional file 1.

**Table 1:** Clinical characteristics of patients according to ethnic group

	**Norwegians**	**Pakistanis**	
	n = 21	n = 18	**p**
Males (%)	10 (48%)	9 (50%)	
Age (years)	42 (6)	41 (8)	0.865
Years with diabetes	5 (9)	9 (7)	**0.021**
Weight (kg)	106.8 ± 13.6	90.1 ±23.4	**0.009**
BMI (kg/m^2^)	37.2 (6.0)	30.9 (9.4)	**0.008**
Waist circumference (cm)	114.3 ± 10.9	106.5 ±17.4	0.102
Waist-hip ratio	1.00 ± 0.09	1.01 ± 0.09	0.575
Total body fat (%)	36.9 ± 9.6	34.2 ± 7.7	0.395
Total body fat mass (kg)	39.5 ± 12.0	28.0 ± 8.2	**0.005**
Lean body mass (kg)	67.1 ± 12.6	54.2 ± 12.2	**0.007**
Fasting plasma glucose (mmol/l)	10.7 ± 3.2	10.6 ± 3.3	0.973
HbA_1c_ (%/mmol/mol)	7.3/56 (1.4/13)	8.7/72 (2.9/31)	**0.022**
Fasting insulin (pmol/l)	166 (160)	209 (193)	0.405
Fasting C-peptide (pmol/l)	1162 ± 458	977 ± 373	0.193
Total cholesterol (mmol/l)	4.5 ± 1.0	4.7 ± 1.3	0.512
HDL cholesterol (mmol/l)	1.03 ± 0.21	1.08 ± 0.24	0.557
LDL cholesterol (mmol/l)	2.7 ± 0.8	2.6 ± 0.6	0.868
Triglycerides (mmol/l)	1.6 (1.1)	1.4 (1.1)	0.832

Table 1: For normally distributed parameters, mean ± SD is given, and comparisons were made using Student’s *t* test. For non-normally distributed parameters, median (interquartile range) is given, and comparisons were made using the Mann–Whitney *U* test. Median HbA_1c_-values are reported as NGSP-%/IFCC-mmol/mol with interquartile range in parentheses. In bold: p < 0.05.

### Ethnic differences in insulin sensitivity

When examining insulin sensitivity with a two-step euglycemic, hyperinsulinemic clamp, median values for GIR and ISI showed consistently higher point estimates in the Norwegian compared to the Pakistani group, although we found no statistically significant crude differences between the groups in GIR or ISI during the first step (Table 2). However, when adjusting for sex and % TBF in a multiple regression analysis, LogGIR_40_ showed a clear tendency towards lower values in the Pakistani group, and LogISI_40_ was significantly lower in the Pakistani compared to the Norwegian group (Table 3). Further adjustment for BMI, HbA_1c_ or diabetes duration did not change the relation of ISI_40_ or GIR_40_ to ethnicity. Ethnicity, sex and % TBF together explained 55% of the variation in insulin sensitivity, expressed as ISI_40_. During the second step of the clamp, Pakistani patients had non-significantly lower median GIR_400_, and significantly lower ISI_400_ (Table 2). In standard multiple regression analyses, ethnicity and % TBF explained 29% of ISI_400_ and 24% of GIR_400_ variation (Table 3). Sex was no longer a significant predictor of LogISI_400_ or Log GIR_400_, and neither was BMI, diabetes duration or HbA_1c_.

**Table 2:** Insulin sensitivity and NEFA suppression

	**Norwegians**	**Pakistanis**	**p**
GIR_40_	468.4 (587.3)	339.8 (468.0)	0.456
ISI_40_	79.7 (137.9)	57.1 (74.1)	0.289
S-insulin _end low step_(pmol/l)	546 (336)	715 (243)	**0.041**
GIR_400_	2055.6 (907.0)	1661.1 (672.3)	0.080
ISI_400_	20.7 (17.2)	14.2 (7.3)	**0.016**
S-insulin _end high step_ (pmol/l)	9505 (2738)	11082 (2922)	0.069
Fasting p-NEFA (mmol/l)	0.59 (0.43)	0.50 (0.29)	0.394
NEFA suppression 0–30 minutes (%)	71.2 (42.1)	41.9 (90.6)	**0.042**

Table 2: Data are presented as median (interquartile range), and comparisons are made using the Mann–Whitney *U* test. In bold: p < 0.05. GIR_40_ = glucose infusion rate_low step,_ ISI_40_ = glucose infusion rate/s-insulin_low step_, GIR_400_ = glucose infusion rate_high step_, ISI_400_ = glucose infusion rate/s-insulin_high step_. GIR is expressed as μmol/m^2^/min, and ISI as 100 × μmol/m^2^/min/s-Ins. NEFA = non-esterified fatty acids. Number of Norwegians and Pakistanis were: during the low step, n = 19 and 16, respectively. During the high step, n = 19 and 14, respectively, and for NEFA measurements, n = 13 and 15, respectively.

**Table 3:** Multiple regression analyses of insulin sensitivity

**Variable**	**Adjusted effect β**	**95% CI**	**p-value**	**Adjusted effect β**	**95% CI**	**p-value**
**Model A:**	**LogISI**_**40**_			**LogGIR**_**40**_		
Ethnicity	-0.335	(-0.588, -0.081)	**0.012**	-0.210	(-0.429, 0.010)	0.060
Sex	-0.726	(-1.129, -0.322)	**0.001**	-0.455	(-0.804, -0.106)	**0.013**
% body fat	-0.061	(-0.084, -0.037)	**<0.001**	-0.044	(-0.065, -0.024)	**<0.001**
Model A + BMI	0.008	(-0.015, 0.031)	0.464	0.002	(-0.017, 0.022)	0.803
Model A + HbA_1c_	-0.009	(-0.095, 0.078)	0.835	-0.038	(-0.111, 0.036)	0.300
Model A + Diabetes duration	-0.001	(-0.028, 0.026)	0.941	0.005	(-0.019, 0.028)	0.697
**Model B:**	**LogISI**_**400**_			**Log GIR**_**400**_		
Ethnicity	-0.186	(-0.333, -0.040)	**0.014**	-0.117	(-0.229, -0.005)	**0.042**
% body fat	-0.009	(-0.017, 0.000)	**0.040**	-0.007	(-0.013, 0.000)	**0.043**
Model B + Sex	-0.163	(-0.394, 0.068)	0.158	-0.081	(-0.262, 0.101)	0.369
Model B + BMI	0.005	(-0.007, 0.018)	0.378	0.005	(-0.004, 0.014)	0.295
Model B + HbA_1c_	0.005	(-0.045, 0.055)	0.829	-0.013	(-0.051, 0.025)	0.493
Model B + Diabetes duration	-0.008	(-0.023, 0.007)	0.304	-0.007	(-0.019, 0.005)	0.231

Table 3: Dependent variables in model A: LogISI_40_ or LogGIR_40._ Constant for Log ISI_40_ = 4.428. R^2^: 0.55. Constant for LogGIR_40_ = 4.464. R^2^: 0.48. Reference group for ethnicity = Norwegians, for sex = women. Variables excluded from final models: BMI, HbA_1c_(NGSP) and diabetes duration.

Dependent variables in model B: LogISI_400_ or LogGIR_400._ Constant for Log ISI_400_ = 1.650. R^2^: 0.29. Constant for LogGIR_400_ = 3.558. R^2^: 0.24. Variables excluded from final models: sex, BMI, HbA_1c_ and diabetes duration. In bold: p < 0.05.

### Ethnic differences in the relation of BMI and waist circumference to insulin sensitivity

**Figure 1:**
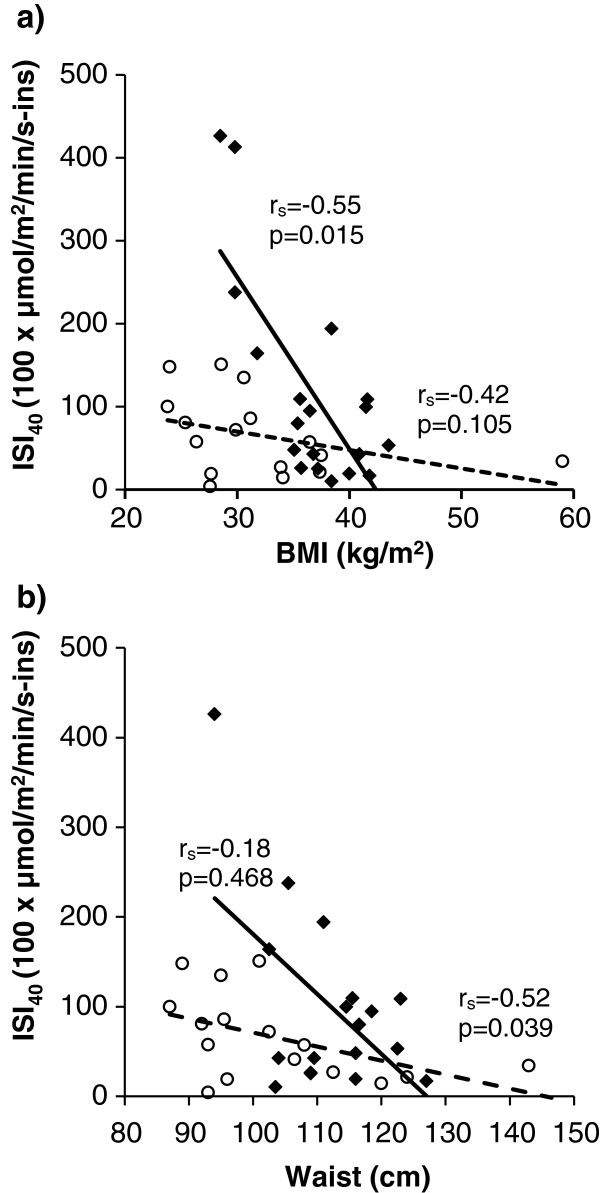
**Relation between ISI**_**40**_**, BMI and waist circumference.** Relation of insulin sensitivity, expressed as ISI_40_, to **a)** body mass index (BMI) and **b)** waist circumference in Norwegian (black diamonds) and Pakistani (white circles) patients with type 2 diabetes. Solid regression lines represent Norwegian patients and dashed lines represent Pakistani patients. Spearman’s Correlation coefficients (r_s_) and p-values for each correlation are presented for both ethnicities. In **a)** the correlations have been performed both including and omitting the outlier (BMI 59). They give approximately the same result, and the outlier is excluded in the r_s_ presented, but shown in the scatterplot.

When exploring the relationships between anthropometric characteristics and insulin sensitivity, we found a strong, significant correlation between ISI_40_ and BMI in the Norwegian group, and a weaker, non significant correlation in the Pakistani group. Waist circumference, however was significantly correlated with ISI_40_ in the Pakistani, but not in the Norwegian group (Figure 1a-b and Additional file 2). There was also a significant interaction between ethnicity and LogBMI *(p = 0.032)* in a multiple regression analysis, suggesting a difference between the ethnic groups. (Dependent variable: LogISI_40_, independent variables: ethnicity *(p = 0,026),* LogBMI *(p = 0,004),* as well as ethnicity x LogBMI). We were, however, not able to confirm an ethnic difference in the relationship between insulin sensitivity and waist circumference by regression analysis (data not shown).

### Ethnic differences in suppression of non-esterified fatty acids

**Figure 2:**
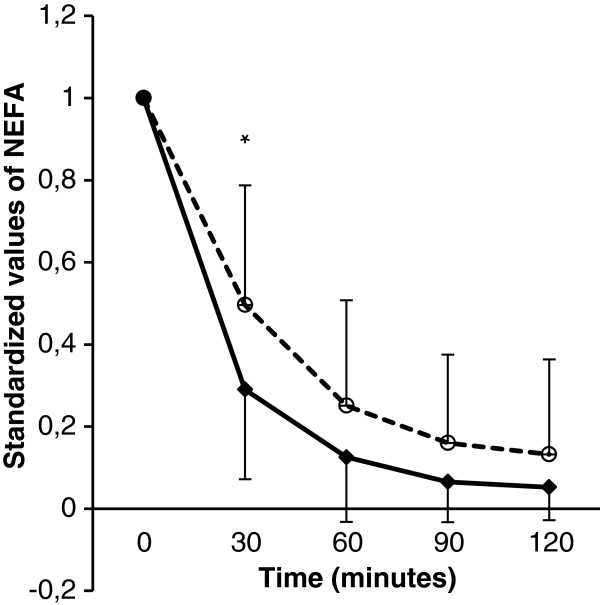
**Suppression of NEFA during euglycemic clamp.** Non-esterified fatty acid (NEFA) values have been standardized, with fasting values set to 1.0. The figure shows standardized mean (SD) values of NEFA during the first 120 minutes of the low step euglycaemic clamp, for both patient groups. Norwegian patients are shown in black diamonds with solid lines. Pakistani patients are shown in white circles with dashed lines. *p < 0.05.

There were no significant differences in fasting NEFA values between Norwegian and Pakistani patients, but initial NEFA suppression by hyperinsulinemia, expressed as percentage suppression of NEFA after 30 minutes of the clamp, was significantly more pronounced in the Norwegian group compared to the Pakistani group (Figure 2, Table 2).

### Adipokines and markers of inflammation

Table 4 shows levels of adipokines and markers of inflammation in the two ethnic groups. The Norwegian, but not the Pakistani group, showed negative correlations between ISI_40_ and leptin, as well as IL-1RA (Additional file 2). Fasting IL-1RA was significantly positively correlated to BMI in both patient groups. In the Norwegian group it was also positively correlated to leptin (r_s_ = 0.59, p = 0.006). In the Pakistani group, IL-1RA neither correlated to ISI, nor to leptin (r_s_ = 0.14, p = 0.593), but correlated positively to waist circumference.

**Table 4:** Adipokines and markers of inflammation

	**Norwegians**	**Pakistanis**	***p*-value**
Adiponectin (μg/ml)	4.6 (4.4)	3.3 (3.4)	0.15
Leptin (pmol/l)	950 (1043)	907 (1014)	0.89
IL-6 (pg/ml)	1.18 (1.19)	1.07 (2.18)	0.43
hsCRP (pg/ml)	2.31 (3.48)	2.45 (2.46)	0.63
IL-1RA (pg/ml)	62.75 (98.10)	45.90 (103.95)	0.36
sTNF-R1 (ng/ml)	1.04 (0.20)	0.92 (0.31)	0.14

Table 4: Data are presented as median (interquartile range), and comparisons are made using the Mann–Whitney *U* test. Nor = Norwegian patients. Pak = Pakistani patients.The number of Nor/Pak were: for adiponectin and leptin, n = 21/18. For IL-6, hsCRP and IL-1RA, n = 20/17, and for sTNF-R1, n = 20/16. IL-6 = interleukin-6, hsCRP = high sensitive C-reactive protein, IL-1RA = interleukin-1 receptor antagonist, sTNF-R1 = soluble tumor necrosis factor-receptor 1.

## Discussion

In the present study we found significant differences in insulin sensitivity, measured by the euglycemic, hyperinsulinemic clamp, between young adult Norwegian and Pakistani patients with T2DM. There were also ethnic differences in diabetes duration and HbA1c, even in this young patient population, demonstrating longer duration of diabetes and poorer glycemic control in the Pakistani group. These differences, however, did not explain the lower insulin sensitivity in the Pakistani group per se, since neither HbA_1c_ nor diabetes duration were significantly associated with the ethnic differences found in insulin sensitivity in adjusted multiple regression analyses.

Furthermore, we demonstrated a different relationship between insulin sensitivity and BMI in the two groups. Although obesity is clearly an important factor in insulin resistance, BMI was a poor marker of metabolic disturbances in the Pakistani patients. Whereas Pakistani patients were all quite insulin resistant regardless of BMI, among the Norwegian patients there was a strong negative correlation between BMI and insulin sensitivity. To our knowledge, this is the first study, using the euglycemic clamp to measure insulin sensitivity, to show such ethnic differences in subjects with T2DM. Our results support and expand previously published results obtained in healthy South Asian immigrants [20,21].

NEFA are normally suppressed by hyperinsulinemia. There is evidence that this suppression is impaired in T2DM [22], although this has not been found by all authors [23]. Our study shows similar fasting NEFA values between the two ethnic groups, but significantly slower NEFA suppression in the Pakistani group, despite higher fasting and end-clamp serum insulin levels. Some evidence of ethnic differences, both in fasting NEFA levels and degree of suppression by hyperinsulinemia, has previously been shown in non-diabetic subjects [24]. Other authors, however, found no difference in NEFA suppression according to ethnicity [25]. Our findings further support that impaired insulin sensitivity affects lipid metabolism more severely in Pakistani patients as compared to Norwegian patients with T2DM.

It is noteworthy that leptin and IL-1RA were differently related to insulin sensitivity in the two ethnic groups. T2DM is now regarded as a disorder characterized by non-resolving inflammation. IL-1, released though activation of Nod-like receptor protein 3 (NLRP3) inflammasomes, seems to play an important role in the inflammatory processes [26]. Il-1RA is considered an anti-inflammatory cytokine, that antagonizes IL-1β and IL-1α, and is elevated, at least in part, in response to elevation of these inflammatory cytokines. An early study showed decreased IL-1RA levels in type 2 diabetes [27], and it has been demonstrated that Leptin decreases β-cell production of IL-1RA, down-regulating IL-1RA expression in pancreatic β-cells in type 2 diabetes [28]. However, more recent studies have shown high levels of IL-1RA in obesity, correlating with BMI, insulin resistance and serum leptin levels, and increased production of IL-1RA in adipose tissue in obese humans [29-31]. Subjects with impaired glucose tolerance have higher levels of IL-1RA [32], and in two prospective cohort studies, IL-1RA was found to be elevated several years before diabetes diagnosis, and significantly predicted incident diabetes [33,34]. Whether this represents a counteracting mechanism in response to IL-1, which circulates at much lower levels in plasma and is more difficult to detect in clinical samples, is at present unclear. In our study, leptin as well as IL-1RA was negatively associated with insulin sensitivity in the Norwegian, but not the Pakistani patients. Leptin and IL-1RA correlated with BMI in both groups, and with waist circumference in the Pakistani group only. These findings, together with the different relationship between insulin sensitivity and BMI demonstrated in our two groups, suggest that there may be important differences in the relationship between obesity, insulin sensitivity, and the effect and/or regulation of these signal molecules between the two ethnic groups. As leptin is known to correlate mainly to superficial abdominal adipose tissue [35,36], our findings could indicate that ethnic differences exist in the metabolic activity of the different compartments of abdominal adipose tissue.

The main strengths of our study include thorough patient examinations with gold standard methods for measurement of insulin sensitivity, and using two steps of hyperinsulinemia. The difference in HbA_1c_ and diabetes duration between the two patient groups may be regarded as a weakness. However, these differences were also present in patients who were not included in the study, and have been demonstrated in several other studies [10-12]. Thus, an attempt to match for these parameters could in our opinion have created a selection bias. It can nevertheless be regarded as a limitation to our study that patients were recruited from a population referred to hospital outpatient diabetes clinics, and that sample size was limited, increasing the risk of type II statistical errors. Recruitment of patients, especially the Pakistani patients, proved challenging, which explains why only 20% of the invited patients participated. While a larger and more representative cohort would have strengthened our study, we note that the ethnic differences in insulin sensitivity showed consistent patterns for all four estimates of insulin sensitivity. This also corresponds well with the clinical picture of higher diabetes prevalence and poorer glycemic control apparent in South Asian populations. We did not measure endogenous glucose production during the euglycemic clamp, and our glucose infusion rates may therefore underestimate the true peripheral glucose uptake, at least during the low insulin infusion level. Lastly, body composition was measured by BIA, which may be less accurate than dual x-ray absorptiometry, computed tomography or magnetic resonance imaging. BIA is, however, an inexpensive and accessible way of estimating body composition, which is gaining use in clinical practice.

## Conclusions

In this study of Norwegian and Pakistani patients with T2DM we found significant differences in insulin sensitivity and the relationship between insulin sensitivity and obesity markers, which may impact on our understanding of the pathogenic mechanisms that place Pakistani subjects at higher risk of developing T2DM. Further studies are needed to elucidate a possible relationship between insulin sensitivity, various adipose tissue compartments and adipokines and related molecules in various ethnic groups.

## Abbreviations

Anti-GAD: Anti-glutamic acid decarboxylate; Anti-IA2: Anti-protein tyrosine phosphatase; BIA: Bioelectrical impedance analysis; BMI: Body-mass index; ELISA: Enzyme linked immunosorbent assay; GIR40: Glucose infusion rate at the low insulin infusion clamp step; GIR400: Glucose infusion rate at the high insulin infusion clamp step; hsCRP: High sensitivity C-reactive protein; IL-1α: Interleukine-1 alpha; IL-1β: Interleukine-1 beta; IL-1RA: Interleukine-1 receptor antagonist; IL-6: Interleukine-6; ISI40: Insulin sensitivity index at the low insulin infusion clamp step; ISI400: Insulin sensitivity index at the high insulin infusion clamp step; NEFA: Non-esterified fatty acids; NLRP3: Nod-like receptor protein 3; RIA: Radio immuno assay; sTNF-R1: Soluble tumor necrosis factor-receptor 1; SD: Standard deviation; T2DM: Type 2 diabetes mellitus; %TBF: Percent total body fat.

## Competing interests

The authors declare that they have no competing interests.

## Authors’ contributions

CW conceived of the study, participated in its design and coordination, researched data and wrote the manuscript. ETA researched data and critically revised the manuscript. TU performed analyses of inflammation markers and contributed to discussion. AEM performed analyses of inflammation markers. PMT participated in the design of the study and revised the manuscript. IFL conceived of the study and participated in patient recruitment. PAT provided the insulin and adipokine analyses and revised the manuscript. PA contributed to discussion and critically revised the manuscript. KIB conceived of the study, participated in its design, contributed to discussion and critically revised the manuscript. All authors read and approved the final manuscript.

## Pre-publication history

The pre-publication history for this paper can be accessed here:

http://www.biomedcentral.com/1472-6823/13/49/prepub

## Supplementary Material

Additional file 1 **Table showing.** Further clinical characteristics of patients according to ethnic group. Click here for file

Additional file 2 **Table showing.** Correlations between parameters of insulin sensitivity, anthropometry, adipokines and inflammation. Click here for file
