# Supplementary material for: Differences in insulin sensitivity, lipid metabolism and inflammation between young adult Pakistani and Norwegian patients with type 2 diabetes: a cross sectional study
Source: BMC Endocr Disord. 2013 Oct 22;13:49. doi: 10.1186/1472-6823-13-49 (PMC4015764; doi:10.1186/1472-6823-13-49)
Supplement: Additional file 1 — Table showing. Further clinical characteristics of patients according to ethnic group. [file 1472-6823-13-49-S1.pdf]

Additional file 1: Further clinical characteristics of patients according to ethnic group.

	**Norwegians**	**Pakistanis**
	n = 21	n = 18
Diabetes treatment n(%)		
Lifestyle ± OAD / Insulin ± OAD	11 (52%) / 10 (48%)	5 (28%) / 13 (72%)
Other medications		
Statins	10 (48%)	4 (22%)
Blood pressure lowering agents	8 (38%)	7 (39%)
Self reported complications		
Macrovascular	2 (10%)	3 (17%)
Retinopathy	2 (10%)	3 (17%)
Nephropathy or microalbuminuria	2 (10%)	6 (33%)
Neuropathy	1 (5%)	3 (17%)
Diabetic foot	1 (5%)	0 (0%)
Others (ED, fatty liver, periodontitis)	5 (24%)	6 (33%)
Co-morbidities		
Astma/COPD	4 (19%)	4 (22%)
Psychiatric conditions	4 (19%)	1 (6%)
GI disease	5 (24%)	2 (11%)
Other endocrine disorders	2 (10%)	3 (17%)
Smoking	10	1
Parity in women		
0-2	11	4
3-5	0	5

Supplementary Table 1: Parity was defined as the number of live-born children each woman had given birth to. OAD = oral antidiabetic drugs. ED = erectile dysfunction. COPD = chronic obstructive pulmonary disease. GI = gastro-intestinal.
