# Supplementary material for: Differences in insulin sensitivity, lipid metabolism and inflammation between young adult Pakistani and Norwegian patients with type 2 diabetes: a cross sectional study
Source: BMC Endocr Disord. 2013 Oct 22;13:49. doi: 10.1186/1472-6823-13-49 (PMC4015764; doi:10.1186/1472-6823-13-49)
Supplement: Additional file 2 — Table showing. Correlations between parameters of insulin sensitivity, anthropometry, adipokines and inflammation. [file 1472-6823-13-49-S2.pdf]

Additional file 2: Correlations between parameters of insulin sensitivity, anthropometry, adipokines and inflammation

	**ISI_{400}**		**BMI**		**Waist**		**% Fat**		**Adiponectin**		**Leptin**		**IL-6**		**hsCRP**		**TNF-R1**		**IL-1RA**	
	Nor	Pak	Nor	Pak	Nor	Pak	Nor	Pak	Nor	Pak	Nor	Pak	Nor	Pak	Nor	Pak	Nor	Pak	Nor	Pak
**ISI40** (r_s)	**0.76**	**0.65**	**-0.55**	-0.42	-0.18	**-0.52**	-0.42	**-0.62**	0.37	-0.06	**-0.52**	-0.16	0.05	0.15	-0.22	-0.31	-0.21	0.31	**-0.72**	0.00
p-values	**<0.001**	**0.017**	**0.015**	0.105	0.468	**0.039**	0.102	**0.024**	0,121	0.837	**0.022**	0.549	0.847	0.589	0.361	0.265	0.384	0.288	**0.001**	0.990
**ISI 400** (r_s)			-0.27	-0.09	-0.15	-0.21	-0.18	**-0.56**	0.35	0.04	-0.39	-0.06	0.07	0.11	-0.07	-0.22	0.12	0.03	**-0.49**	-0.16
p-values			0.270	0.759	0.570	0.474	0.516	**0.047**	0.139	0.887	0.098	0.840	0.773	0.697	0.791	0.455	0.632	0.929	**0.039**	0.603
**BMI** (r_s)					**0.54**	**0.92**	**0.62**	0.41	-0.15	0.04	**0.68**	**0.56**	0.02	0.03	-0.17	0.28	0.36	-0.23	**0.50**	**0.49**
p-values					**0.017**	**<0.001**	**0.008**	0.144	0.551	0.874	**0.001**	**0.016**	0.925	0.900	0.487	0.273	0.116	0.386	**0.026**	**0.045**
**Waist** (r_s)							0.17	0.34	-0.10	-0.08	0.27	**0.48**	-0.12	0.03	-0.14	0.33	0.37	-0.19	0.30	**0.625**
p-values							0.520	0.230	0.681	0.760	0.264	**0.042**	0.637	0.911	0.567	0.202	0.125	0.478	0.206	**0.007**
**% Fat** (r_s)									-0.21	0.37	**0.82**	**0.86**	-0.06	0.08	-0.28	0.33	0.25	-0.50	0.48	-0.08
p-values									0.411	0.196	**<0.001**	**<0.001**	0.829	0.793	0.294	0.245	0.343	0.085	0.058	0.799

Supplementary table 2: Spearman's correlation coefficients (r_s) with p-values. Correlations with $p<0.05$ are shown in bold. Nor=Norwegian patients. Pak=Pakistani patients. ISI_{40}=glucose infusion rate/s-insulin$_{low\ step}$, ISI_{400}=glucose infusion rate/s-insulin$_{high\ step}$. BMI=body mass index. Waist=waist circumference. % Fat=percentage total body fat. IL-6=interleukin-6, hsCRP=high sensitive C-reactive protein, sTNF-R1=soluble tumor necrosis factor-receptor 1, IL-1RA=interleukin-1 receptor antagonist.
